# Antenatal diagnosis and management of pregnancy luteoma: A case report and literature review

**DOI:** 10.1097/MD.0000000000034521

**Published:** 2023-07-28

**Authors:** Junhua Shen, Jingyi Li, Xia Tao, Yan Feng, Baohua Li

**Affiliations:** a Department of Obstetrics, Women’s Hospital, Zhejiang University School of Medicine, Hangzhou, P.R. China; b Zhejiang Provincial Clinical Research Center for Obstetrics and Gynecology, Hangzhou, P.R. China; c Department of Ultrasonography, Women’s Hospital, Zhejiang University School of Medicine, Hangzhou, P.R. China.

**Keywords:** antenatal diagnosis, intrauterine growth restriction, pregnancy luteoma, prenatal hyperandrogenization

## Abstract

Pregnancy luoteomas are tumor-like ovarian lesions that emerge during pregnancy and spontaneously regress after delivery. Antenatal diagnosis is infrequently reported, and unnecessary surgery appears to be common in literature reports.

**Case summary:**

A 28-year-old primigravida with bilateral adnexal masses was discovered at 32 + 5 weeks during prenatal ultrasound evaluation. Combined with clinical presentation, auxiliary examinations including blood test, magnetic resonance imaging, gastroscopy, and consultation of multi-disciplinary team, we successfully made a diagnosis of pregnancy luteoma and provided conservative management recommendations. A cesarean section was conducted on this patient at 34 + 2 weeks of gestation due to fetal distress. The newborn was small for gestational age but normal in appearance. We performed biopsies of the adnexal masses, which were confirmed to be pregnancy luteomas using both intraoperative frozen section and final pathological diagnosis. Serum testosterone, cancer antigen 125, and alpha-fetoprotein levels gradually declined and normalized on postoperative day 28. The masses significantly decreased in size as shown by ultrasonic and magnetic resonance imaging examination on postoperative day 7, with the ovaries returning to their normal size by postoperative day 30.

**Conclusion:**

Prenatal diagnosis of pregnancy luteoma poses a challenge, requiring hormonal examinations, ultrasound, magnetic resonance imaging, and gastrointestinal endoscopy for identification. Caution must be exercised to avoid overtreatment. While additional cases are needed to summarize the imaging features and effects of excess hormones on the both mother and fetus, further research is necessary for a comprehensive understanding.

## 1. Introduction

Pregnancy luteomas were first described by Dr William Sternberg in 1963, they are rare tumor-like lesions of the ovary that emerge during pregnancy and regress spontaneously after delivery. These masses can be unilateral or bilateral, solid in consistency, may reach up to 20 cm in diameter, and are typically asymptomatic and incidentally discovered during cesarean section or postpartum tubal sterilization.^[1]^

The factors of morbidity, pathogenesis, and their potential influences on pregnancy remain unclear. Using the search terms “pregnancy luteoma,” we conducted a comprehensive literature review from 2000 to the present on PubMed and Web of Science databases, resulting in the identification of 25 reports encompassing 26 cases of pregnancy luteomas^[2–26]^ (Table 1). To summarize the literature, it is noteworthy that achieving an accurate prenatal diagnosis poses a significant challenge. Some reports have described luteoma detection occurring incidentally during routine ultrasonography antepartum,^[2–5,7,9,12–14,16,18,19,21–23]^ in such cases, when the ultrasound and magnetic resonance imaging (MRI) results suggested a diagnosis of ovarian neoplasia, a laparotomy was offered in order to rule out malignancy.^[2,7,12–14,18,19,22,23]^ In addition, partial ovariectomy or oophorectomy remained the commonly performed procedures, in these instances, the biopsy ought to have sufficed and preservation of the ovaries should have been prioritized.^[2–4,6–10,12–22,24,25]^ In the present study, we present a complete case of pregnancy luteoma including antenatal diagnosis and management strategy. Written consent was obtained from all patients. Based on a thorough literature review and multidisciplinary team consultation, we accurately diagnosed antenatal pregnancy luteoma, and made an informed decision to prolong the gestational period and avoid excessive surgery. Our antenatally diagnosed case and experiences may contribute to a better understanding of the natural history and management of pregnant women with luteomas.

**Table 1:** Pregnancy luteoma case reports: literature review from 2000 to present.

Year	Author	Diagnostic Approach	ART/HA/HDCP/IUGR	Management methods/pathological diagnosis	Fetal outcome
2000	Choi et al^[2]^	1. US+T 2. US	1. HA 2.N	1. Spontaneous preterm labor at 29 wk/N 2. Laparotomy and left salpingo-oophorectomy at 21 wk/Y	1. Normal appearance female infant 2. Not mentioned
2002	Mazza et al^[3]^	US+T	HA	US follow-up, bilateral ovarian lesions resection at the cesarean/Y	Female infant with complete masculinization
2003	Wang et al^[4]^	US+MRI	N	US and MRI follow-up, bilateral ovarian lesions resection at the cesarean/Y	Normal appearance female infant
2005	Wang et al^[5]^	US+MRI+T	HA	Vaginal delivery/N	Female infant with clitoral hypertrophy
2006	Banerjee et al^[6]^	N	ART	Left ovarian lesions resection at the cesarean/Y	Normal appearance male infant
2007	Spitzer et al^[7]^	US+MRI+T	ART+HA+HDCP+IUGR	Laparotomy and right salpingo-ophorectomy with omentectomy postpartum/Y	Virilized female infant
2008	Dahl et al^[8]^	US+MRI+T	HA+HDCP	Cesarean, cystic-appearing ovaries untreated/N	Normal appearance male infant
2008	Tan et al^[9]^	US	ART	Laparotomy and right salpingo-oophorectomy/Y	Norma appearance male infant
2009	Wang et al^[10]^	US+T	ART+HA	Not mentioned/Y	Normal appearance male infant
2009	Ugaki et al^[11]^	US	N	US follow up, left ovarian lesions resection at the cesarean/Y	Virilized female infant
2009	Tannus et al^[12]^	US+MRI+T	HA	Laparotomy and right ovarian lesions resection at 22 wk/Y	Normal appearance male infant
2010	Masarie et al^[13]^	US+MRI+T	HA	Laparotomy, cesarean, and right oophorectomy at 37 wk/Y	Normal appearance female infant
2013	Dasari et al^[14]^	US+T	HA	Laparotomy and ovarian biopsy at 8 wk/Y	Miscarried at 17 wk
2014	Nanda et al^[15]^	N	N	Bilateral partial oophorectomy at the cesarean/Y	Normal appearance female infant
2014	Ranjan et al^[16]^	US	N	Left salpingo-oophorectomy at the cesarean/Y	Normal appearance infant
2014	Wadzinski et al^[17]^	US+MRI	ART+HA+HDCP	Caesarean section but ovarian lesions not mentioned/N	Twin female infants with virilization
2016	Limaiem et al^[18]^	US	N	Laparotomy and left salpingo-oophorectomy/Y	Not mentioned
2016	Rapisarda et al^[19]^	US+T	HA	US follow up, laparotomy, cesarean and right oophorectomy at 34 wk/Y	Normal appearance male infant
2016	Verma et al^[20]^	N	N	Right oophorectomy at the cesarean/Y	Normal appearance female infant
2017	Brar et al^[21]^	US	N	Laparotomy and left ovarian lesions resection/Y	N(ectopic pregnancy)
2017	Khurana and O’Boyle^[22]^	US+MRI	N	Laparotomy and mass resection at 23 wk/Y	Full term birth and normal-looking infant
2017	Rathore et al^[23]^	US	N	Laparotomy and salpingo-oophorectomy/Y	N(ectopic pregnancy)
2019	Smolarczyk et al^[24]^	T	HA	Resection of the right ovary and partial resection of the left ovary/Y	Normal appearance female infant
2021	Mvunta et al^[25]^	N	N	Left unilateral oophorectomy at the cesarean/Y	Normal appearance female infant
2022	Zhu et al^[26]^	US+CT	ART	Exploratory laparotomy and salpingo-oophorectomy due to torsion/Y	Not mentioned
2023	Our case report	US+MRI+T+Gastroscopy	HA+IUGR	Ovarian biopsy at the cesarean/Y	Normal appearance male infant

ART = assisted reproductive technology, CT = computerized tomography, HA = hyperandrogenism, HDCP = hypertensive disorder complicating pregnancy, IUGR = intrauterine growth restriction, MRI = magnetic resonance imaging, N = no or not mentioned, T = testosterone test, US = ultrasonography, Y = yes.

## 2. Case presentation

**Figure 1.**
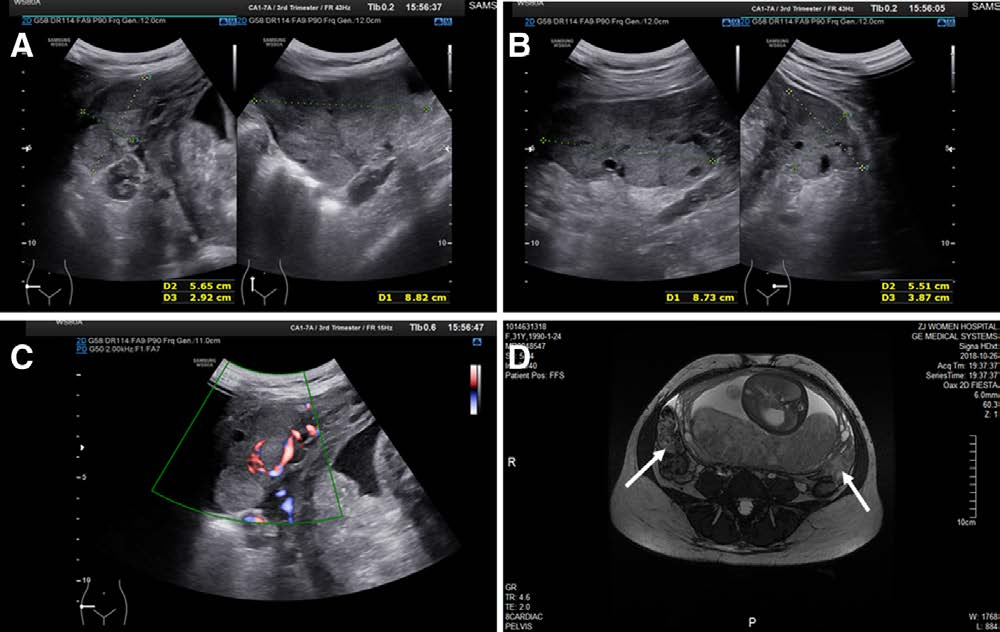
Sonography and magnetic resonance imaging of the ovarian masses. (A) Sonography of left maternal adnexal mass about 8.7 × 5.5 × 3.9 cm. (B) Sonography of right maternal adnexal mass about 8.8 × 5.7 × 2.9 cm. (C) Color Doppler evaluation demonstrated prominent arterial and venous flows. (D) The MR images show bilateral adnexal mass, mainly solid, but many vesicles were visible. MR = magnetic resonance.

A 28 years old primigravida was referred to our center from a local hospital for routine prenatal ultrasound, which suggested intrauterine growth restriction at 32 + 5 weeks of gestation. She told us that for about 1 month, she experienced both deepening of her voice and persistent facial acne. She conceived naturally, and her prenatal serological screening indicated a high-risk due to a free-β-human chorionic gonadotropin (hCG) elevation of 424 ng/mL (multiples of the median value 26.27). Her non invasive prenatal genetic testing results were at low risk. Her menarche had occurred at 14 years of age, with menses generally being regular, and her past medical history was uneventful. Physical examination revealed a short uterine height of 28 cm and dark coarse hair on her anterior abdomen. Her body mass index was 23.1 kg/m^2^ and blood pressure was within the normal range. A fetal ultrasonography demonstrated a fetus with biparietal diameter of 8.1 cm, head circumference 29.3 cm, femur length 5.5 cm, and abdomen circumference 25.1 cm. In addition, bilateral adnexal masses were incidentally found, presenting as solid and with a left mass size about 8.7 × 5.5 × 3.9 cm and right mass size about 8.8 × 5.7 × 2.9 cm, with prominent arterial and venous flow (Fig. 1). The right iliac fossa also had a free-fluid depth of 3.9 cm. Laboratory tests were notable for fasting plasma glucose elevated to 6.35 mmol/L (normal < 5.0), C-reactive protein elevated to 19.8 mg/L (normal < 5.0), a cancer antigen 125 (CA125) of 96.8 U/mL (normal < 35), and alpha-fetoprotein (AFP) of 205.1 ng/mL (normal < 7). Hormone determination tested as abnormal with a testosterone level of 65.0 nmol/L (normal < 3.0) and hCG of 137,381.0 IU/L (normal < 5.0).

We also reviewed patients’ prior ultrasound scans. Systemic ultrasound at 12 and 22 weeks of gestation showed normal fetal growth and no adnexal masses. An initial multi-disciplinary team discussion was called, comprising obstetricians, gynecologic oncologists, neonatologists, pathologists, radiologists, and medical directors to discuss the source of the newly found adnexal masses. The conclusion was that for this rapidly increasing mass, we primarily needed to consider a possible source of malignancy, combined with hyperandrogenemia, may be ovarian sex cord stromal tumors, metastatic ovarian tumors, or adrenal tumors. However, it was also noted that pregnancy luteomas or hyperreactive luteinization couldn’t be ruled out, but were highlighted as rarely occurring. The subsequent MRI, detailed the left 8.5 × 5.6 × 3.8 cm, right 8.6 × 5.5 × 3.0 cm solid adnexal mass with small internal vesicles (Fig. 1), and confirmed no obvious fetal structural abnormalities, maternal adrenal glands abnormalities, and no abnormally enlarged lymph nodes in the pelvic and abdominal cavities. The patient also underwent a gastrointestinal endoscopy, the result was a diagnosis of chronic gastritis. Subsequently, a second multi-disciplinary team discussion was conducted. The conclusion was that this mass was likely benign rather than malignant, conservative management including closely monitored fetal intrauterine growth and ovarian masses development was recommended. We had a detailed communication with the gravida and her husband, who assented to our management suggestions, and written consent was obtained from the patient.

**Figure 2.**
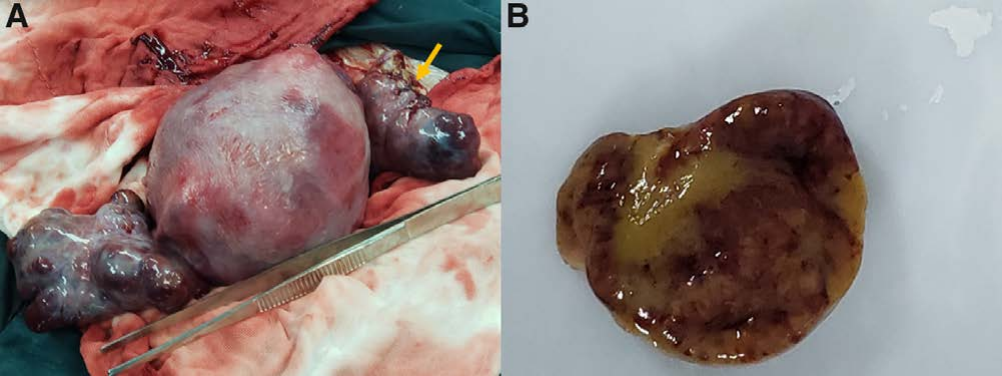
Intraoperative findings. (A) A tissue section of about 1 × 1 × 1 cm was sampled from the mass and the wound was sutured for bleeding (yellow arrow). (B) The section shows brown tissue without necrosis.

**Figure 3.**
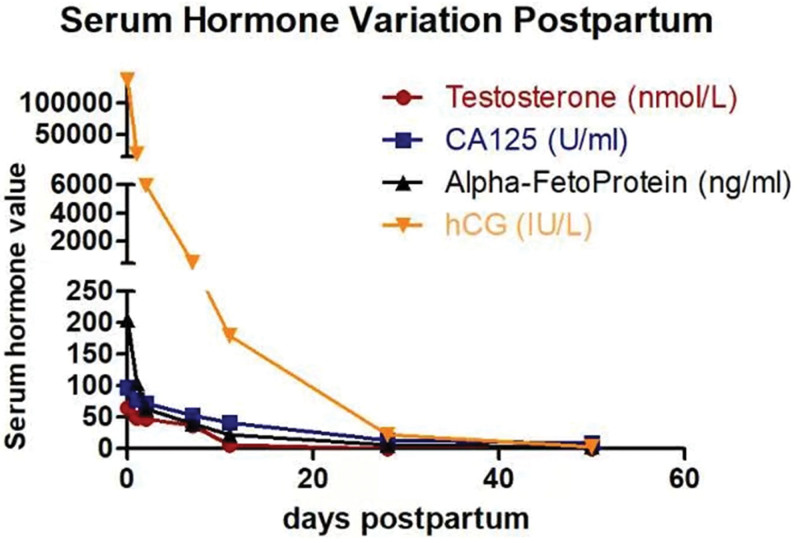
Serum hormone, AFP, and CA125 variation postpartum. AFP = alpha-fetoprotein, CA125 = cancer antigen 125.

The patient underwent fetal heart rate monitoring every day, due to fetal distress, we performed a cesarean section at 34 + 2 weeks of gestation. A normal appearance male newborn was delivered, his weight was 1570 g, and APGAR scores were 10-10 at 5 to 10 minutes. Severe intrauterine growth restriction (IUGR) was defined as birth weight less than the 3th percentile. Intraoperative exploration revealed bilateral, crineous, solid lesions on the ovarian surfaces, with the external surface prominently bosselated. During the operation, abscission cytology of the ascites was conducted and a tissue section of about 1 × 1 × 1 cm was cut for intraoperative frozen sectioning (Fig. 2), and intraoperative pathological report confirmed “microscope conform pregnancy luteoma.” We then decided to preserve the lesions and sent the placenta for pathological examination. The final pathological result confirmed the diagnosis of pregnancy luteoma, with placental pathological results noting that “placental villi were sparsely developed.” Cytological results of ascites exfoliation indicated no signs of tumor cells. Monitoring of serum testosterone, CA125, and AFP showed a decrease at an approximate speed, all normalized at 28 days post-operation (Fig. 3). Ultrasound imaging revealed a reduction in the size of the left ovarian mass to 5.2 × 3.4 × 3.1 cm and right ovarian mass to 5.2 × 4.0 × 3.6 cm on postoperative day 7, followed by normalization of bilateral ovaries with no abnormal echogenicity observed on ultrasound and MRI at 30-day follow-up. The patient reported lactation initiation 2 days post-operatively and was discharged 3 days later. During the 1-year follow-up, she reported a decrease in hirsutism, normalization of voice and menstruation, and normal growth and development of her infant.

## 3. Discussion and conclusion

The true incidence of pregnancy luteomas is unknown, but it is widely believed that they occur more commonly than currently diagnosed.^[1,6,10,13,17]^ Owing to emerging improvements in the technologies related to prenatal imaging examination, increasing numbers of pre-delivery diagnoses are likely to be forthcoming. Antenatal diagnosis is rarely reported, and in reviewing this case, we consider that we made a correct antenatal diagnosis of pregnancy luteoma. Newly discovered solid masses with rich blood supply and pelvic effusion, combined with elevated tumor markers, gave us an initial suspicion of a malignant tumor. However, maternal virilization was a key factor that helped us switch the diagnosis to pregnancy luteoma. During pregnancy, pelvic mass with hyperandrogenemia should promote the consideration of ovarian sex cord stromal tumors, Sertoli-Leydig cell and Krukenberg tumors, adrenal tumors and ovarian tumor-like lesions of luteomas or hyperreactio luteinalis.^[5,7,17,27,28]^ However, as tumor markers such as AFP and CA125 may be expressed by healthy fetal tissues and detected in considerable concentrations in maternal serum, their value in the assessment of malignant disease in pregnant women is somewhat limited.^[29]^ MRI findings of pregnancy luteomas require a larger sample size to provide a comprehensive summary of their characteristic features. Despite this, MRI examinations are useful for ruling out adrenal tumors and fetal anomalies and also serve as an effective tool for identifying lymph node metastases. Although gastrointestinal endoscopies have not been mentioned in other case studies, we believe that this method is advantageous given past instances where gastrointestinal neoplasms were mistakenly diagnosed as pregnancy luteomas.^[30–33]^ Additionally, utilizing gastrointestinal endoscopy for diagnostic purposes continues to be a secure procedure during gestation.^[34]^

The etiology of pregnancy luteomas remains unknown. The term “hCG dependent” has been used in some previous studies to describe this condition,^[7,10,13,22]^ however, we were unable to find any specific data regarding the role of hCG over the recent 20 years of pregnancy luteoma reports. It could be coincidental that in our case, we found high hCG levels in the second and third trimesters, which suggests that the high hCG state was sustained throughout pregnancy. In addition, the reported cases contained no associations with twins or trophoblastic diseases, conditions in which hCG levels would be abnormally elevated. It could be that hCG is not consistently high enough, or that high hCG may simply be a stimulating factor in the formation of pregnancy lutiomas, but not highly notable as a singular factor. Similarly, our patient exhibited a significant elevation in testosterone levels. It has been reported that hyperandrogenemia is present in 25% to 65% of pregnancy luteomas.^[13,35,36]^ While not directly demonstrated on a pathological level, it was generally believed that the origin of this hyperandrogenism appeared to be ovarian and that androgen production was observed in proportion to the size of the ovarian mass.^[28]^ Despite increases in testosterone, an increase in sex hormone-binding globulin and the activation of placental aromatase cytochrome P450 may reduce excess androgen exposure in both the mother and fetus.^[35]^ However, when excess androgen production by the mother or fetus exceeds the capacity of these protective measures, the functional capacity of aromatase would then be exceeded, which may lead to hyperandrogenemia. In our case, we wondered why testosterone declined so rapidly after the operation since the vast majority of the mass was not removed, but was left to gradually shrink down in size on its own. We therefore considered if the hyperplasia of luteinized stromal cells in pregnancy luoteomas maybe not the only source of hyperandrogenism, but that its hormonal activity may be activated via pregnancy-related hormones such as hCG, or hyperandrogenemia as has been subsequently associated with placental dysfunction. However, this hypothesis remains to be tested.

It is possible that the patient was fortunate to have a male baby. In the surveyed literature, if the fetus of the virilized mother was female, genital anomalies were frequently observed, accounting for 2-thirds of such instances.^[7]^ Correspondingly, the discussions in such reports regarding the impact of pregnancy luteomas on pregnancy have primarily focused on high androgen levels affecting fetal gonadal development. However, there have been limited reports on the effects of this condition on other pregnancy-related diseases when considering comorbidities. We observed some cases of pregnancy luteomas accompanied by hypertension,^[7,8,17]^ but their relationship was not elaborated. There have been no reports on the relationship between pregnancy luteomas and IUGR. Our study found that fetus had severe IUGR. Several studies have shown that hCG play specialized roles in promoting angiogenesis in the uterine endothelium, maintaining myometrial quiescence, and fostering immunomodulation at the maternal-fetal interface.^[37]^ However, a drop in hCG levels after the second trimester is required for normal pregnancy progression.^[38]^ Some evidence suggests that elevated hCG levels during middle and late pregnancy are associated with preeclampsia.^[39,40]^ Additionally, excess androgen may also affect fetal growth by affecting placental growth and function. Animal studies have provided evidence to support the effect of excess androgen levels on IUGR. Excess prenatal testosterone has been shown to alter nutrient transfer in rodent models^[41]^ and affect placental differentiation in sheep.^[42]^ The above mechanism may be involved in IUGR occurrence in our cases, although IUGR is a syndrome with heterogeneous etiology and a spectrum of phenotypes. Moreover, prenatal hyperandrogenization induces a pro-inflammatory and unbalanced oxidative state in the uterus reflected by increased COX-2, lipid peroxidation, and NF-κB.^[43,44]^ This hypothesis may also explain why we found that the patient had elevated fasting blood glucose and C-reactive protein levels, which did not seem to have an adequate clinical explanation at the time.

This conclusion gave rise to another consideration, as other studies have suggest pregnancy luteomas are recommend to be observed without surgery during pregnancy. Would antiandrogen therapy be a favorable choice especially for those pregnancy luteomas that occur together with high testosterone levels? Future studies focusing on the mechanism of hyperandrogenemia and high hCG levels in pregnancy luteinomas may be helpful for the decision whether to intervene earlier to improve maternal and infant outcome.

There are no guidelines for the optimal management of pregnancy luteoma. We presented a complete case of pregnancy luteoma including complete history, antenatal diagnosis, management strategy and postpartum follow-up. This is an important case, particularly considering that its incidence is widely believed to be underestimated, that it may be easily misdiagnosed or over-treated, and that the accompanying hormonal abnormalities may cause adverse maternal and pregnancy outcomes. We hope that our approach to clinical diagnosis and treatment will bring obstetricians a better awareness of the disease. We still require an increased number of cases to establish a clear framework for the standard understanding of the occurrence and development processes of this disease and its impact on maternal and fetal health and wellbeing.

## Acknowledgement

The authors would like to thank Chris Wood for this discussion.

## Author contributions

**Formal analysis:** Junhua Shen.

**Project administration:** Junhua Shen, Baohua Li.

**Software:** Xia Tao.

**Supervision:** Yan Feng.

**Writing – original draft:** Junhua Shen.

**Writing – review & editing:** Junhua Shen, Jingyi Li, Baohua Li.
